# Supplementary material for: A comparative framework for convergence analysis of perturbation series techniques in nonlinear fractional quadratic differential equations
Source: PLoS One. 2025 Dec 29;20(12):e0337884. doi: 10.1371/journal.pone.0337884 (PMC12747413; doi:10.1371/journal.pone.0337884)
Supplement: Attachment — Submitted filename: Review of the paper PONE-D-25-50040.docx [file pone.0337884.s001.docx]

I have reviewed your paper " A Comparative Framework for Convergence Analysis of Perturbation Series Techniques in Nonlinear Fractional Quadratic Differential Equations" and I have some notes and suggestions as the following:

- The abstract is clear, but it could be shortened.
- In page 3 some symbols have been written in two way, such as p is inconsistently written as $\rho$ and p in this paper.
- Recommended adding more practical, examples from physical or engineering.
- Remove the duplicates from the references, [1] and [4] are duplicates.
- Standardizing the way of references are written must choose one format.
- Finally this paper introduced a distinguished scientific contribution in the field of fractional differential equations.
