# Supplementary material for: A comparative framework for convergence analysis of perturbation series techniques in nonlinear fractional quadratic differential equations
Source: PLoS One. 2025 Dec 29;20(12):e0337884. doi: 10.1371/journal.pone.0337884 (PMC12747413; doi:10.1371/journal.pone.0337884)
Supplement: Attachment — Submitted filename: Fractional Quadratic Differential Equations.docx [file pone.0337884.s002.docx]

" A Comparative Framework for Convergence Analysis of Perturbation Series Techniques in Nonlinear Fractional Quadratic Differential Equations"

# 1) The abstract is clear, but it could be shortened.

# 2) In page 3 some symbols have been written in two way, such as p is inconsistently written as $\rho$ and p in this paper.

# 3) Recommended adding more practical, examples from physical or engineering.

# 4) Remove the duplicates from the references, [1] and [4] are duplicates.

# 5) Standardizing the way of references are written must choose one format.

# 6) Finally this paper introduced a distinguished scientific contribution in the field of fractional differential equations.
