# Supplementary material for: A comparative framework for convergence analysis of perturbation series techniques in nonlinear fractional quadratic differential equations
Source: PLoS One. 2025 Dec 29;20(12):e0337884. doi: 10.1371/journal.pone.0337884 (PMC12747413; doi:10.1371/journal.pone.0337884)
Supplement: Attachment — Submitted filename: Response to Reviewers.docx [file pone.0337884.s003.docx]

Response to Reviewers

Plos one Journal

Manuscript Number : PONE-D-25-50040

A Comparative Framework for Convergence Analysis of Perturbation Series Techniques in Nonlinear Fractional Quadratic Differential Equations

Corresponding author: Dulfikar Jawad Hashim

Comments of Reviewers:

| No | Comment | | Correction |
| --- | --- | --- | --- |
| 1 | | The abstract is clear, but it could be shortened | The abstract has been carefully shortened while maintaining its academic value and preserving the essential objectives, methodology, and main findings of the study |
| 2 | | In page 3 some symbols have been written in two way, such as p is inconsistently written as $\text{ρ}$ and p in this paper. | P refers to embedding parameter for homotopy methods, while $\text{ρ}$ refers to fractional derivatives, To avoid any confusion, we will use a different symbol for the fractional derivative $\text{(}\text{ℬ}\text{)}$ in all equations of the manuscript. |
| 3 | | Recommended adding more practical, examples from physical or engineering. | A second example involving the second-order fractional Riccati equation has been added. This example demonstrates the method’s applicability to problems that depend on higher-order fractional derivatives and have several practical applications. |
| 4 | | Remove the duplicates from the references, [1] and [4] are duplicates. | The duplicate references ([1] and [4]) have been corrected, and a more relevant source has been added. |
| 5 | | Standardizing the way of references are written must choose one format. | All references have been reviewed and standardized according to a single format throughout the manuscript. |
| 6 | | Finally this paper introduced a distinguished scientific contribution in the field of fractional differential equations. | We sincerely thank the reviewer for the positive feedback and acknowledgment of our contribution. |

Notes:

(1) : Several additional references have been included to strengthen the credibility of the manuscript. Moreover, all references have been reformatted to comply with the journal’s template requirements.
(2) : We sincerely appreciate the valuable comments provided by the reviewers, which greatly helped us improve the quality of the manuscript.
